# Microarray Analysis of *Campylobacter Jejuni*: To the Guts of the Problem!

**DOI:** 10.1002/cfg.185

**Published:** 2002-08

**Authors:** Nick Dorrell, Olivia L. Champion, Brendan W. Wren

**Affiliations:** 1 Department of Infectious and Tropical Diseases London School of Hygiene and Tropical Medicine London WC1E 7HT UK; 2 Pathogen Molecular Biology and Biochemistry Unit Department of Infectious and Tropical Diseases London School of Hygiene and Tropical Medicine London WC1E 7HT UK


**Comparative and Functional Genomics**
*Comp Funct Genom* 2002; **3**: 338–341.
Published online in Wiley InterScience (www.interscience.wiley.com). **DOI:** 10.1002/cfg.185

**Conference Review**

### Microarray analysis of *Campylobacter jejuni*: to the guts of the problem!

Nick Dorrell,* Olivia L. Champion and Brendan W. Wren
*Department of Infectious and Tropical Diseases, London School of Hygiene and Tropical Medicine, London WC1E 7HT, UK*

**Correspondence to:
Nick Dorrell, Pathogen Molecular Biology and Biochemistry Unit, Department of Infectious and Tropical Diseases, London School of Hygiene and Tropical Medicine, London WC1E 7HT, UK.
E-mail: nick.dorrell@lshtm.ac.uk*

Received: 31 May 2002
Accepted: 7 June 2002

### The first BμG@S microarray

*Campylobacter jejuni* is recognized as the leading cause of food-borne bacterial diarrhoeal disease throughout the world [1]. In the UK it is predicted that there are over half a million cases a year. *Campylobacter* enteritis is a more protracted illness than the *Salmonella* equivalent (5–10 days compared with 24 h), so the cost to the UK economy through lost working days is significant. Furthermore, infection with this bacterium has been implicated as a frequent antecedent to the development of the neurological diseases Guillain-Barré syndrome and Miller Fisher syndrome [5]. The natural hosts for *C. jejuni* are avian species, where the bacterium is a commensal organism and does not cause disease. Thus, *C. jejuni* readily colonizes chickens and other poultry and thereby enters the food chain. However, despite intensive study, including the publication of the genome sequence of strain NCTC 11168 in February 2000 [6], there have been few advances in the development of control and intervention strategies and the mechanism(s) by which *C. jejuni* causes diarrhoeal disease are still unclear.

A *C. jejuni* NCTC 11168 whole genome microarray was the first array constructed at the BμG@S facility. The first generation *C. jejuni* array utilized the ordered plasmid library that was a by-product of the genome sequencing project (performed at the Sanger Institute, Cambridge, UK) by selecting an optimum clone to represent each gene in the genome and amplifying PCR products from these selected clones using a single pair of vector primers [2]. This approach was selected because it was inexpensive compared to purchasing oligonucleotide primers to amplify gene-specific PCR products and to validate microarray technology with respect to bacterial pathogens. This low-cost microarray contained 34.5% gene-specific probes, whilst the remainder contained either single (35.4%) or multiple (30.1%) adjacent gene fragments. Our aim has been to exploit this plasmid clone array for both comparative genomic and gene expression studies with *C. jejuni*.

### Microarray-based epidemiology?

A comparative genomics study of 11 human isolates revealed extensive genetic diversity between

Copyright © 2002 John Wiley & Sons, Ltd.

these *C. jejuni* strains [2]. Many of the strain-variable genes are associated with the biosynthesis of surface structures, including flagella, lipo-oligosaccharide and capsule. This suggests that variation of these determinants may be important in survival, transmission and pathogenesis, indicating that selective pressures have driven profound evolutionary changes to create a diverse *Campylobacter* species. Comparison of the capsule biosynthesis locus reveals conservation of all the genes in this region in strains with the same Penner serotype as strain NCTC 11168. By contrast, between five and 17 NCTC 11168 genes in this region are either absent or highly divergent in strains of a different serotype to the sequenced strain, providing further evidence that the capsule accounts for Penner serotype specificity. In all, at least 21% of the genes present in the sequenced strain appear dispensable, as they are absent or highly divergent in one or more of the isolates tested, defining 1300 out of 1654 genes as *C. jejuni* species-specific. These genes mainly encode housekeeping functions, such as metabolic, biosynthetic, cellular and regulatory processes. However, many virulence determinants are also conserved, indicating that they are indispensable for *C. jejuni* to cause disease in humans. These include the cytolethal distending toxin, the flagellar structural proteins, the PEB antigenic surface proteins and the general protein glycosylation locus.

*C. jejuni* strain diversity combined with variable host responses results in a complex spectrum of disease outcomes, ranging from a symptomatic colonization to severe inflammatory diarrhoea. A major reason for continued comparative genomics studies is the absence of an animal model that reflects *C. jejuni*-associated disease. Precise strain comparisons from well-characterized strains of diverse origins may allow correlates of pathogenesis to be determined and the subsequent identification of potential virulence determinants. Also, an understanding of genetic differences between *C. jejuni* strains from different ecological niches should allow the identification of improved epidemiological markers and the development of rational approaches to reduce this problematic pathogen from the food chain.

In addition to investigating human clinical isolates of diverse origins, current projects are also attempting to investigate differences in genomic content of *C. jejuni* strains from a wider variety of sources. For example, multilocus sequence typing (MLST) of *C. jejuni* strains has identified two clonal complexes associated with the sand of bathing beaches, and not with human disease cases, livestock or chickens (K.E. Dingle, personal communication). It is thought that the natural host of these isolates may be wild birds, and that faecal contamination of the beaches by birds preceded the isolation of these strains, as a single isolate from this source is placed in one of these complexes. We predict that these strains are missing genes that prevent them from causing human disease or colonizing chickens. Using microarray analysis to identify genes that are absent or highly divergent in these clonal complexes may allow us to identify novel genes with an important role in human virulence or chicken colonization. Another interesting set of strains has been identified from the recent Infectious Intestinal Diseases study by the Foods Standards Agency, where some of the control patients were found to be carrying *C. jejuni* but were asymptomatic. Microarray analysis of such strains could highlight genes that may have a role in causing human diarrhoeal disease.

### Accurate transcription analysis?

In addition to comparative genomic studies, we have also carried out gene expression-based studies. One of our main considerations has always been to obtain meaningful data, and an important contributing factor to this is the isolation of total RNA that contains an mRNA population that represents the condition under investigation, rather than conditions involved in the RNA isolation protocol. Both high-speed centrifugation and lysing bacterial cell walls are steps in current commercial RNA isolation kits that will result in changes in gene expression before mRNA stabilization. The use of high concentrations of guanidine isothiocynate for immediate chemical stabilization of mRNA, as pioneered for RNA isolation from *Mycobacterium* species [4], is not possible for *C. jejuni*, as the bacterial cells lyse under the highly denaturing conditions. Therefore, we developed a protocol to minimize RNA isolation protocol-induced changes in *C. jejuni* gene expression. This incorporates a short low-speed centrifugation of a 50 ml culture

Copyright © 2002 John Wiley & Sons, Ltd. 

volume to quickly pellet a proportion of the bacterial cells, followed by a rapid disruption of the cells and mRNA stabilization using the Hybaid Ribolyser™ kit. The RNA is further purified using the Qiagen RNeasy Mini™ kit to produce total RNA suitable for microarray analysis. Additionally, we always isolate our test and control RNA samples at the same time, as a matched pair, to avoid the effect of any day-to-day variation in the protocol. Using this protocol, we routinely isolate 50 μg total RNA at a concentration of $>1$ μg/μl, which is necessary to perform microarray hybridizations in triplicate from the same sample. Comparing gene expression profiles between the *C. jejuni* NCTC 11168 wild-type strain and a *wlaC* mutant has validated this approach. WlaC is involved in protein glycosylation (post-translational modification) and thus mutation of the *wlaC* gene was predicted to not affect gene expression. Microarray analysis has shown that less than 10 genes appear to be up- or downregulated in this mutant, suggesting that our RNA isolation protocol produces mRNA, which reflects the conditions under test rather than the isolation protocol.

Currently we are investigating the effect of changes in culture conditions on the gene expression profiles of the wild-type *C. jejuni* NCTC 11168 strain. Changes in culture conditions aim to mimic environmental stresses that the bacteria will encounter. For example, the body temperature of avian species (the natural host for *C. jejuni*) is 42 °C and the bacteria grow optimally at this temperature, rather than at 37 °C. An obvious experiment was to compare gene expression profiles at the two temperatures; however, microarray analysis has revealed relatively few changes in gene expression at 42 °C, with the most significant occurring in metabolic pathways. For example, microarray analysis indicates that the succinate dehydrogenase operon (*sdhABC*) is downregulated at 42 °C. Succinate dehydrogenase is a constituent of the tricarboxylic acid cycle, but the biochemical significance of downregulation at 42 °C is unclear. Another area of investigation has been the response of *C. jejuni* to bile salt stress, which is a relevant *in vivo* stress that *C. jejuni* must survive. We have studied gene expression changes in the presence of the bile salt deoxycholate at 37 °C and microarray analysis indicates a greater change in gene expression. The most interesting changes appear to affect the surface structures of the bacteria and these are currently under further investigation.

As well as varying the culture conditions to induce changes in gene expression, comparing isogenic defined mutants against the wild-type strain can give important clues to gene function. However, as shown with the *wlaC* mutant mentioned above, not all mutations will have a dramatic effect on gene expression, so careful selection of mutants is required to maximize the information that microarray analysis can provide. Therefore, our initial studies have concentrated on strains with defined mutations in known regulatory genes, such as alternate sigma factors and two-component regulators. Microarray analysis of such regulatory gene mutants can confirm known roles for the gene product in the control of gene expression (and thus partially validate the microarray data) and also reveal previously unknown functions. For example, we have performed microarray analysis with defined *fliA*, *rpoN* and *flgR* mutants, three genes known to play a role in the regulation of flagellar biosynthesis [3]. This has confirmed and expanded our understanding of the regulation of gene expression during flagellar biosynthesis, but also highlighted the regulatory roles these genes play in flagellar-independent pathways, which are now also under further investigation.

### The new hope?

The low-cost microarray used so far in our studies has given us an earlier insight into microarray analysis of *C. jejuni* than would otherwise have been possible. However, there are drawbacks with this type of plasmid clone array. Not all the reporter elements are gene-specific, so the presence of adjacent genes may increase the amount of hybridization from the sample and thus increase the signal intensity recorded, producing inaccurate data. Also the melting temperatures of the reporter elements are variable, resulting in uneven hybridization profiles at any given temperature. A gene-specific *C. jejuni* array with reporter elements designed using the BμG@S minimal cross-hybridization protocol (which also designs PCR products with similar melting temperatures) has now been constructed, which also includes genes absent in the NCTC 11168 genome. This second-generation array begins the move away from a

Copyright © 2002 John Wiley & Sons, Ltd.

strain-specific array to a more representative array of the *C. jejuni* species. Currently a second *C. jejuni* strain (RM1221) is being sequenced at TIGR [7] and genes present in this strain but absent in NCTC 11168 will be included on a third-generation array.

Microarray analysis of *C. jejuni* using both comparative genomic and gene expression analysis approaches is allowing us to rapidly increase our understanding of this enigmatic human pathogen. We anticipate major steps forward in disease control and intervention strategies, based on a greater knowledge of the ecology, epidemiology and pathogenesis of this organism. This should enable us to get to the guts of the problem sooner rather than later!!

### Acknowledgements

We acknowledge BμG@S (the Bacterial Microarray Group at St George's) and the Wellcome Trust for funding their work, and Aparna Jagannathan and Charles Penn for the collaboration on flagellar biosynthesis. We also thank the BBSRC, the MRC and the Wellcome Trust for financial support.

### References

1. Blaser MJ. 1997. Epidemiologic and clinical features of *Campylobacter jejuni* infections. *J Infect Dis* **176**(Suppl 2): S103–S105.
2. Dorrell N, *et al.* 2001. Whole genome comparison of *Campylobacter jejuni* human isolates using a low-cost microarray reveals extensive genetic diversity. *Genome Res* **11**: 1706–1715.
3. Jagannathan A, *et al.* 2001. Roles of rpoN, fliA, and flgR in expression of flagella in *Campylobacter jejuni*. *J Bacteriol* **183**: 2937–2942.
4. Monahan IM, *et al.* 2001. Extraction of RNA from intracellular *M. tuberculosis*; methods, considerations and applications. In *Methods in Molecular Medicine: Mycobacterium tuberculosis*, Parish T, Stoker NG (eds). Humana Press: Totowa, NJ; 31–42.
5. Nachamkin I, *et al.* 1998. *Campylobacter* species and Guillain-Barré syndrome. *Clin Microbiol Rev* **11**: 555–567.
6. Parkhill J, *et al.* 2000. The genome sequence of the food-borne pathogen *Campylobacter jejuni* reveals hypervariable sequences. *Nature* **403**: 665–668.
7. TIGR Microbial database 'In Progress' page: **http://www.tigr.org/tdb/mdb/mdbinprogress.html**

Copyright © 2002 John Wiley & Sons, Ltd. 
